# Canine *DVL2* variant contributes to brachycephalic phenotype and caudal vertebral anomalies

**DOI:** 10.1007/s00439-021-02261-8

**Published:** 2021-02-18

**Authors:** Julia E. Niskanen, Vilma Reunanen, Milla Salonen, Danika Bannasch, Anu K. Lappalainen, Hannes Lohi, Marjo K. Hytönen

**Affiliations:** 1 Department of Medical and Clinical Genetics, University of Helsinki, Helsinki, Finland; 2 Folkhälsan Research Center, Helsinki, Finland; 3 Department of Veterinary Biosciences, University of Helsinki, Helsinki, Finland; 4 Department of Equine and Small Animal Medicine, University of Helsinki, Helsinki, Finland; 5 Department of Population Health and Reproduction, University of California Davis, Davis, CA 95616 USA

## Abstract

A frameshift deletion variant in the Wnt pathway gene dishevelled 2 (*DVL2*) is associated with a truncated, kinked tail (“screw tail”) in English Bulldogs, French Bulldogs and Boston Terriers. These breeds are also characterized by distinctive morphological traits, including a wide head, flat face and short-limbed dwarfism, which are characteristic of Robinow syndrome in humans, caused by defects in genes such as *DVL1* and *DVL3*. Based on these phenotypic and genetic similarities, it has previously been hypothesized that the canine *DVL2* variant results in a syndromic phenotype called the Robinow-like syndrome. In our study, we investigated the distribution of the *DVL2* variant in 1954 dogs from 15 breeds, identifying breeds with allele variation and enabling the dissection of the genotype–phenotype correlation for the first time. With CT examinations in American Staffordshire Terriers, we confirmed that the *DVL2* allele is associated with caudal vertebral malformations and a brachycephalic phenotype. We also hypothesize that the variant may be linked to additional health conditions, including brachycephalic obstructive airway syndrome and congenital heart defects. Altogether, our study strengthens the role of *DVL2* as one of the contributors to the “bulldog type” morphology and features on the spectrum of human Robinow syndrome.

**Supplementary Information:**

The online version contains supplementary material available at 10.1007/s00439-021-02261-8.

## Introduction

The domestic dog (*Canis lupus familiaris*) exhibits tremendous morphological variety due to breed propagation. Recently, Mansour et al. (2018) showed that caudal vertebral malformations, or a shortened and kinked tail (also referred to as a “screw tail”), are a consequence of a frameshift deletion variant in the *DVL2* (dishevelled segment polarity protein 2) gene. Screw tail is a distinctive trait in English Bulldogs, French Bulldogs, and Boston Terriers. These breeds are also characterized by other vertebral anomalies, brachycephalic skull morphology, widely set eyes, and short stature. Similar clinical signs are seen in Robinow syndrome in humans, a hereditary disorder caused by gene defects in the Wnt signaling pathway, including *DVL1*, *DVL3, ROR2*, *WNT5A*, *FZD2* and *NXN* (White et al. 2018). Robinow syndrome is characterized by distinctive facial features, e.g., prominent forehead, widely spaced eyes and a flat nasal bridge; mesomelic limb shortening; and variable cardiac, oral and urogenital anomalies (Soman and Lingappa 2015). Based on the genetic and clinical similarities between Robinow patients and these breeds, Mansour et al. (2018) suggested that the *DVL2* variant could be responsible for the “bulldog type” morphology and contribute to a Robinow-like syndrome in dogs.

The *DVL2* variant segregates in a recessive manner with caudal vertebral malformations and has incomplete and variable penetrance for thoracic vertebral malformations (Mansour et al. 2018). However, since the allele is entirely or nearly fixed in English Bulldogs, French Bulldogs and Boston Terriers (Mansour et al. 2018), it has been unclear what the variant’s effects are on other morphological features and whether heterozygosity causes any phenotype. Our study investigated the deletion allele distribution in these three breeds, confirming that the variant is fixed, and identified breeds with allele variation, which allowed us to dissect the effects of the allele on canine morphology.

## Materials and methods

### Study cohort and DNA extraction

EDTA blood samples were collected from a total of 1954 privately owned dogs: 165 Boston Terriers, 297 French Bulldogs, 211 English Bulldogs, 4 Olde English Bulldogges, 11 American Bulldogs, 285 American Staffordshire Terriers (AST), 714 Staffordshire Bull Terriers (SBT), 73 Dogues de Bordeaux, 16 Bull Terriers, 13 Miniature Bull Terriers, 40 Lhasa Apsos, 20 Shih Tzus, 46 Tibetan Spaniels, 10 Pekingese, 47 King Charles Spaniels and 2 mixed breed dogs (French Bulldog × German Shepherd Dog). Genomic DNA was extracted with a semi-automated Chemagic 360 extraction robot (PerkinElmer Chemagen Technologie GmbH, Germany). DNA concentration was measured with Nanodrop ND-1000 UV/Vis Spectrophotometer (Nanodrop technologies, Wilmington, Delaware, USA) or DeNovix DS-11 Spectrophotometer (DeNovix Inc., Wilmington, Delaware, USA). The samples were stored at − 20 °C. Sample collection was approved by the Animal Ethics Committee of the State Provincial Office of Southern Finland (ESAVI/343/04.10.07/2016 and ESAVI/25696/2020).

### Variant screening

We genotyped the *DVL2* variant in the 1954 dogs with standard PCR and Sanger sequencing using the primers designed by Mansour et al. (2018): 5′-CGGCTAGCTGTCAGTTCTGG-3′ (forward) and 5′-CAGTGAGTCTGAGCCCTCCA-3′ (reverse). The amplified PCR products were sequenced with a capillary sequencer (Applied Biosystems ABI3730XL DNA Analyzer) at the Institute for Molecular Medicine Finland (FIMM) core facility. The sequences were analyzed using the Sequencher (version 5.3, GeneCodes, USA) and UGENE software (Okonechnikov et al. 2012).

### Prospective CT imaging

We recruited nineteen ASTs (11 males and eight females) with known *DVL2* genotypes (eight wild types, eight heterozygotes, and three homozygotes) to a full-body CT examination at the University of Helsinki Veterinary Teaching Hospital. A physical examination was performed, body weight and age were recorded, and height at the withers was measured using measuring tape with bubble level.

Each dog was mildly sedated for the CT scan with 0.002–0.006 mg/kg dexmedetomidine (Dexdomitor; Orion, Finland) and 0.08–0.2 mg/kg butorphanol (Butordol; Intervet International B.V., Boxmeer, Netherlands) administered intramuscularly (IM) as a single injection. According to two dog owners, one dog had a mild aortic stenosis and another had an innocent murmur, both diagnosed by echocardiography. In these two cases, a veterinary anesthesiologist was consulted prior to sedation.

A non-contrast full-body helical CT imaging was performed in dorsal recumbency with GE LightSpeed VCT 64 (GE Healthcare, Fairfield, Connecticut). The parameters used were 120 kV tube voltage, maximum current 750 mAs, noise index 10, interval 0,625 mm, 0,625 mm slice thickness in both bone and soft tissue algorithms (bone window: center 500, width 1500 Hounsfield units; soft tissue window: center 40, width 400 Hounsfield units), using a 512 × 512 matrix. The acquisition extended from the tip of the nose to the tip of the tail.

In addition to the nineteen ASTs, we also recruited one wild-type SBT to a CT examination due to a short and kinked tail. CT imaging, physical examination and sedation were performed as described above.

### Radiographs

Spinal radiographs of twenty-three ASTs (12 males, 11 females) with known *DVL2* genotype (21 wild types, 1 heterozygote, 1 homozygote) were retrieved from the Finnish Kennel Club’s image database with the owner’s informed consent and evaluated and measured retrospectively for this study. The set consisted of laterolateral images of cervical, thoracic and lumbar spine with the sacrum, and a ventrodorsal image of the pelvis. One dog also had laterolateral and ventrodorsal images of the tail. Age, gender, and body weight were also recorded.

As a pilot study, spinal and tail radiographs of three mixed breed dogs (French Bulldog × German Shepherd Dog) from the same litter were taken at the University of Helsinki Veterinary Teaching Hospital. The physical examination and sedation were performed as described above.

### Assessment and measurements of the images

All images were interpreted by a clinical instructor with ten years of experience in radiology (VR), unaware of the dogs’ *DVL2* genotypes. CT image analysis was done using Horos software (v2.0.0 RC3 open-source PACS workstation DICOM viewer). Radiographs were assessed using Clear Canvas (Synaptive Medical, Toronto, Canada, version 7.0) PACS workstation DICOM viewer.

From the CT images, hemivertebrae were classified and all other vertebral malformations recorded as previously described (Gutierrez-Quintana et al. 2014). The presence of spinal degenerative changes and spondylosis deformans, calcified discs and any other abnormalities were also recorded. The vertebral segment was recorded for each vertebral malformation and for each patient. Transitional vertebrae were not counted in the number of caudal vertebral malformations. All thoracic, lumbar and sacral vertebrae were measured from multiplanar sagittal reformatted images. The maximum length of the dorsal and ventral halves of the vertebral body were recorded. The maximum height of the vertebral body was measured in the cranio-caudal midpoint. The length of the bony tail was measured, and the number of caudal vertebrae was counted.

The length of the radial axis was measured bilaterally from dorsal multiplanar reformatted images. Joint orientation lines were drawn along the proximolateral aspect and the proximomedial aspect of the radial head and along the distolateral aspect and the distomedial aspect of the distal radius (Fox et al. 2006). The length of the radius was measured between those lines.

Cephalometric measurements included facial length and width, cranial length and width, mandibular length, skull length and width and skull base length (Evans and de Lahunta 2013). Distance between the eyes was measured as the distance between the right and left frontolacrimal suture, nasal bone length was measured from the nasion to the rostral end of os nasale midline and hard palate length from caudal to the rostral end of the hard palate. Soft palate length and thickness were measured on sagittal view as previously published (Heidenreich et al. 2016). Finally, the following indices were calculated: skull index = skull width × 100/skull length, cranial index = cranial width × 100/cranial length, and facial index = skull width × 100/facial length (Evans and de Lahunta 2013).

From the radiographs, vertebral malformations were classified, measured and recorded similarly to the CT images. The length of the bony tail was measured from the laterolateral image of the dog with radiographs of the tail.

### Statistical analyses

The association of the *DVL2* genotype with body measurements was examined with linear models and generalized linear models. The data consisted of the same 19 ASTs that underwent CT examination. We examined the association of the dog’s genotype with 11 measurements, ratios and indices: body weight, height at the withers, radius length, soft palate length, soft palate height, hard palate length, skull base length, ratio of hard palate length to skull base length, facial index, cranial index and skull index. These variables were explained with the dog’s *DVL2* genotype (wild type/heterozygote/homozygote) and sex. Dog’s body weight was also included as an explanatory variable if this improved the model fit, as evaluated by a decrease of at least 2 units of the Akaike Information Criterion (AIC). This resulted in the inclusion of body weight only in the soft palate height model. Length of the radius of both the right and left front leg was measured and thus, we built a mixed model with genotype and sex as fixed variables and dog identity as a random variable.

Model fit was assessed thoroughly. First, different distributions and link functions were compared by fitting the model and visually examining the residuals with packages boot and rcompanion (Davison and Hinkley 1997; Canty and Ripley 2019; Mangiafico 2019). Based on visual examination, linear models were used in analyses of body weight, height at the withers, radius length (linear mixed model), soft palate length, hard palate length, skull base length, cranial index and skull index. For ratio of hard palate length to skull base length and facial index, we built generalized linear models with gamma distribution and log link function. For soft palate height, inverse gaussian distribution with a log link function provided the best fit. Second, after choosing the distribution and link function, outliers were examined and plotted with packages broom, dplyr and ggplot2 (Wickham 2016; Robinson and Hayes 2019; Wickham et al. 2019). Third, multicollinearity was evaluated with variance inflation factor (VIF) using the package car (Fox and Weisberg 2019). Finally, the linearity of the continuous explanatory variable, body weight, was assessed by fitting a generalized additive model with the package gam (Hastie 2019).

To estimate the overall effect of variables, an analysis of variance (ANOVA) was run with the package car (Fox and Weisberg 2019). To obtain mean estimates for categorical variables and pairwise comparisons between levels of categorical variables, we calculated the estimated marginal means with the package emmeans (Lenth 2019). The estimates for the continuous variable were obtained with the package effects (Fox 2003; Fox and Weisberg 2019). Due to a high number of analyses and pairwise comparisons, all *p*-values were controlled for false discovery rate (FDR). The significance cut-off value was set to *p* < 0.05. All statistical analyses were conducted with R version 3.6.2 (R Core Team 2019).

## Results

### Prevalence of the *DVL2* variant

We genotyped the *DVL2* variant in English Bulldogs, French Bulldogs and Boston Terriers to investigate the prevalence of the deletion allele in a large cohort. To identify breeds with *DVL2* allele variation and enable the investigation of the phenotype-genotype correlation, we also screened other breeds in which the allele segregates (Mansour et al. 2018) and closely related breeds. Furthermore, King Charles Spaniels were included due to a high prevalence of significant caudal vertebral anomalies (Hytönen et al. 2009). In total, the cohort included 1954 dogs (Table 1).

**Table 1:** Frequency of the *DVL2* deletion allele in the studied breeds

Breed	wt/wt (*N*)	wt/del (*N*)	del/del (*N*)	Total (*N*)	wt/del (%)	del/del (%)
Boston Terrier*	0	0	165	165	0	100
French Bulldog*	0	0	211	211	0	100
English Bulldog*	0	0	297	297	0	100
American Staffordshire Terrier (AST)	201	79	5	285	27.7	1.8
Staffordshire Bull Terrier (SBT) *	578	135	1	714	18.9	0.1
Dogue de Bordeaux	46	27	0	73	37.0	0
Olde English Bulldogge	2	2	0	4	50.0	0
American Bulldog	9	2	0	11	18.2	0
Bull Terrier	16	0	0	16	0	0
Miniature Bull Terrier	13	0	0	13	0	0
Lhasa Apso	40	0	0	40	0	0
Shih Tzu*	20	0	0	20	0	0
Tibetan Spaniel	46	0	0	46	0	0
Pekingese	10	0	0	10	0	0
King Charles Spaniel	47	0	0	47	0	0
Mixed breed (French Bulldog/German Shepherd Dog)	0	2	0	2		
Total	1028	247	679	1954		

Breeds that have previously been found to carry the allele (Mansour et al. 2018) are denoted with an asterisk (*)

The *DVL2* variant was completely fixed in Boston Terriers, French Bulldogs and English Bulldogs. Deletion homozygotes and heterozygotes were additionally discovered in ASTs and SBTs. Furthermore, heterozygotes were found in Dogues de Bordeaux, Olde English Bulldogges and American Bulldogs. Overall, the carrier frequency in these five breed cohorts ranged from 18.2% to 50.0%; however, sample sizes were highly variable (from 4 to 708). Finally, the variant was absent in the seven other breeds.

### Clinical findings

To dissect the effect of the *DVL2* deletion on canine morphology, we carried out full-body CT examinations (Online material 1, Suppl. Tables 1–5) and retrospectively assessed radiographic images in a cohort of dogs with known genotypes (Online material 2, Suppl. tables 6–8). We chose to perform the examinations on ASTs because, among breeds with allele variation, the number of homozygous dogs was the highest. Nineteen dogs (11 males, eight females) participated in the CT examinations. The mean age of the cohort was 5.7 years (min: 2.7, max: 10.6, SD: 2.8), and the average weight was 29.2 kg in males (min: 23.0, max: 36.5, SD: 3.3) and 25.2 kg in females (min: 21.4, max: 29.2, SD: 2.5). Among the 19 dogs, 3 were homozygous, 8 were heterozygous and 8 were wild-type for the *DVL2* variant.

**Fig. 1:**
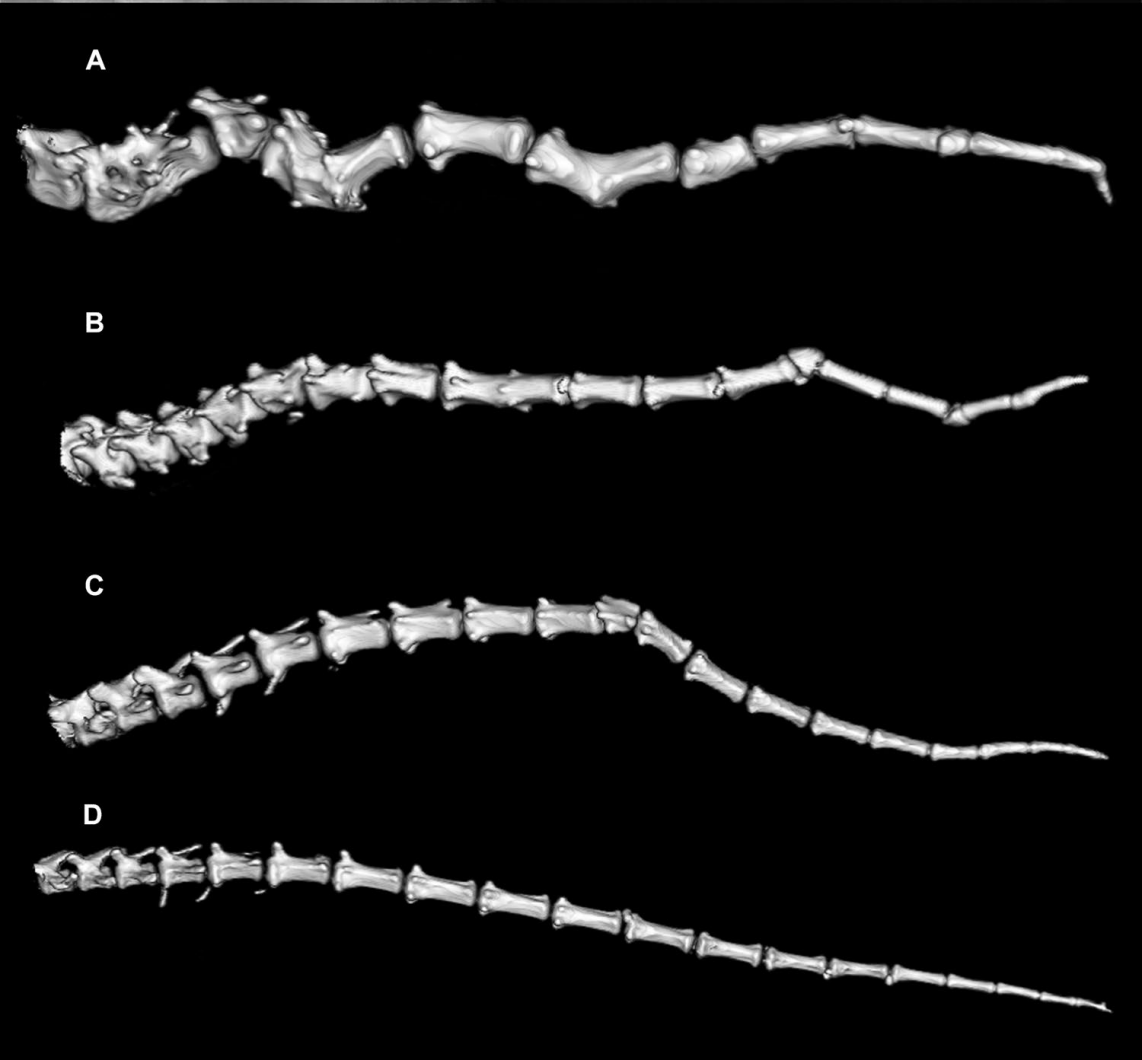
Volume rendering technique (3D) computed tomography images of tails of four American Staffordshire Terriers. **a**–**c** are *DVL2* homozygous dogs with varying number and types of vertebral malformations. The dog in image A with most malformations had also abnormal sacral vertebrae. **d** Normal tail of a *DVL2* wild-type dog

The tail could be completely evaluated in all of the CT imaged dogs and in the radiographs of one *DVL2* homozygote (*N* = 20). In the *DVL2* homozygous dogs (*N* = 4), mean tail length was 26 cm, which was below the mean of heterozygous (33 cm, *N* = 8) and wild-type (34 cm, *N* = 8) dogs. The total number of caudal vertebrae ranged from 19 to 21 in heterozygous and wild-type dogs and from 20 to 21 in homozygous dogs, all of which had abnormal vertebrae in the tail (Fig. 1). In the *DVL2* homozygotes, the number of malformed caudal vertebrae per dog was 1, 8, 10 and 14, respectively. One or several of the following caudal vertebral malformations were detected: block vertebra, dorso-lateral hemivertebra, lateral hemivertebra, butterfly vertebra, ventral wedge shape, unclassified congenital malformation and abnormally short vertebra. Additionally, one homozygote had vertebral malformations in the sacrum, namely ventral wedge shape vertebra and an unclassified congenital malformation. No other vertebral malformations were detected in these four homozygous dogs. Finally, the owners of two homozygous dogs unavailable for a clinical examination (one AST and one SBT) reported that their dogs’ tails were kinked.

**Fig. 2:**
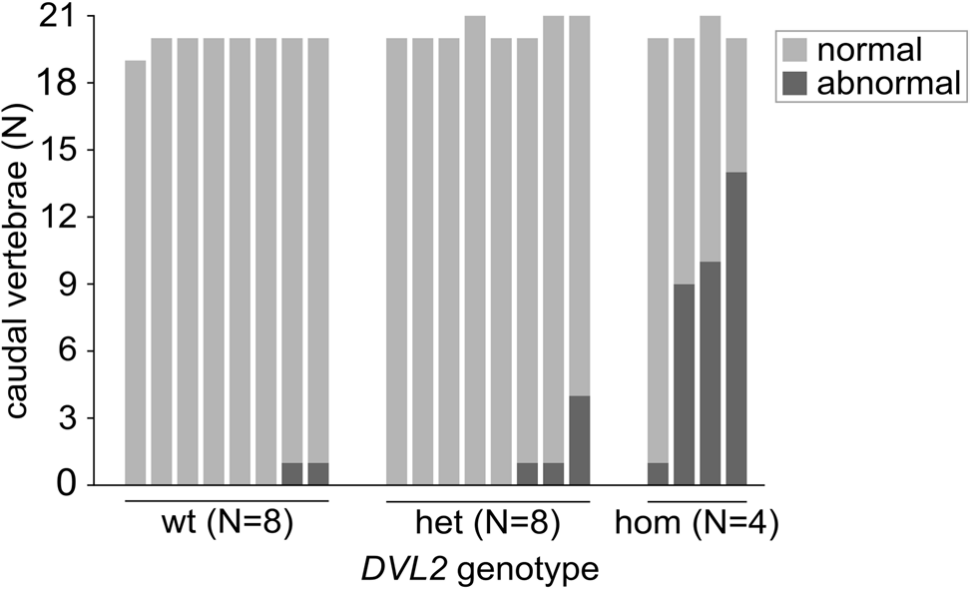
The number of normal and abnormal caudal vertebrae in by *DVL2* genotype in one radiographed and nineteen CT examined American Staffordshire Terriers

In addition to the *DVL2* homozygotes, several heterozygous and wild-type dogs with tail anomalies were discovered in CT examinations and in the rest of the screening cohort (Fig. 2, Table 2). First, three out of the eight CT examined *DVL2* heterozygotes exhibited caudal vertebral malformations: one had four malformations (ventral wedge shape and dorsal hemivertebra and two unclassified congenital malformations) and two dogs had one malformation (unclassified congenital malformation). Second, two out of the eight CT examined wild-type ASTs had one caudal vertebral malformation (dorso-lateral hemivertebra and unclassified congenital malformation). Additionally, one CT examined wild-type SBT had a short tail consisting of only 11 caudal vertebrae, which is less than the average of 20 caudal vertebrae typically seen in dogs with normal tails such as our wild-type ASTs. The penultimate vertebra had an unclassified congenital malformation, and the last vertebra was small, triangular and pointing dorsally. The other caudal vertebrae were normal. Thoracic, lumbar or sacral vertebral malformations were not detected in any *DVL2* heterozygotes or wild types.

**Table 2:** The number of tail anomalies in heterozygous and wild-type dogs observed in CT examinations or reported by the owner

Breed	Source	Dogs with tail anomalies (*N*)		Screening cohort (*N*)
		het	wt	
American Staffordshire Terrier (AST)	CT examination	3	2	280
	Owner report	1	0	
Staffordshire Bull Terrier (SBT)	CT examination	NA	1	713
	Owner report	5	8	
Dogue de Bordeaux	Owner report	2	2	73
Olde English Bulldogge	Owner report	1	0	4
American Bulldog	Owner report	0	0	11
	Total	12	13	1081

*Het* heterozygous, *wt* wild-type

In total, 12 heterozygous and 13 wild-type dogs in the screening cohorts had tail anomalies either observed in CT studies or according to the owner’s report (Table 2). Notably, accurate health information was not available for the entire screening cohorts, so the frequency of tail anomalies is not representative of the entire breeds and possibly an underestimation. Finally, all three French Bulldog × German Shepherd crosses had normal spines and normal-length tails without any vertebral malformations.

Shape of the cervical, thoracic and lumbar vertebrae and the ratio of the height to the length of the thoracic and lumbar vertebrae were normal in all dog groups. Spondylosis and features of transitional vertebra were found among all *DVL2* genotypes.

Finally, two of the three *DVL2* homozygotes (one male and one female) in the CT study had asymmetrical frontal sinuses and a mild heart murmur in clinical examination. One dog had previously been diagnosed with aortic stenosis in echocardiography and the other had had echocardiographic measurements within the normal range, but a follow-up examination by a cardiologist had been recommended. Additionally, one homozygous AST unavailable for CT examination had a mild, asymptomatic heart murmur according to the owner’s report.

### Association of the *DVL2* genotype with body measurements

**Fig. 3:**
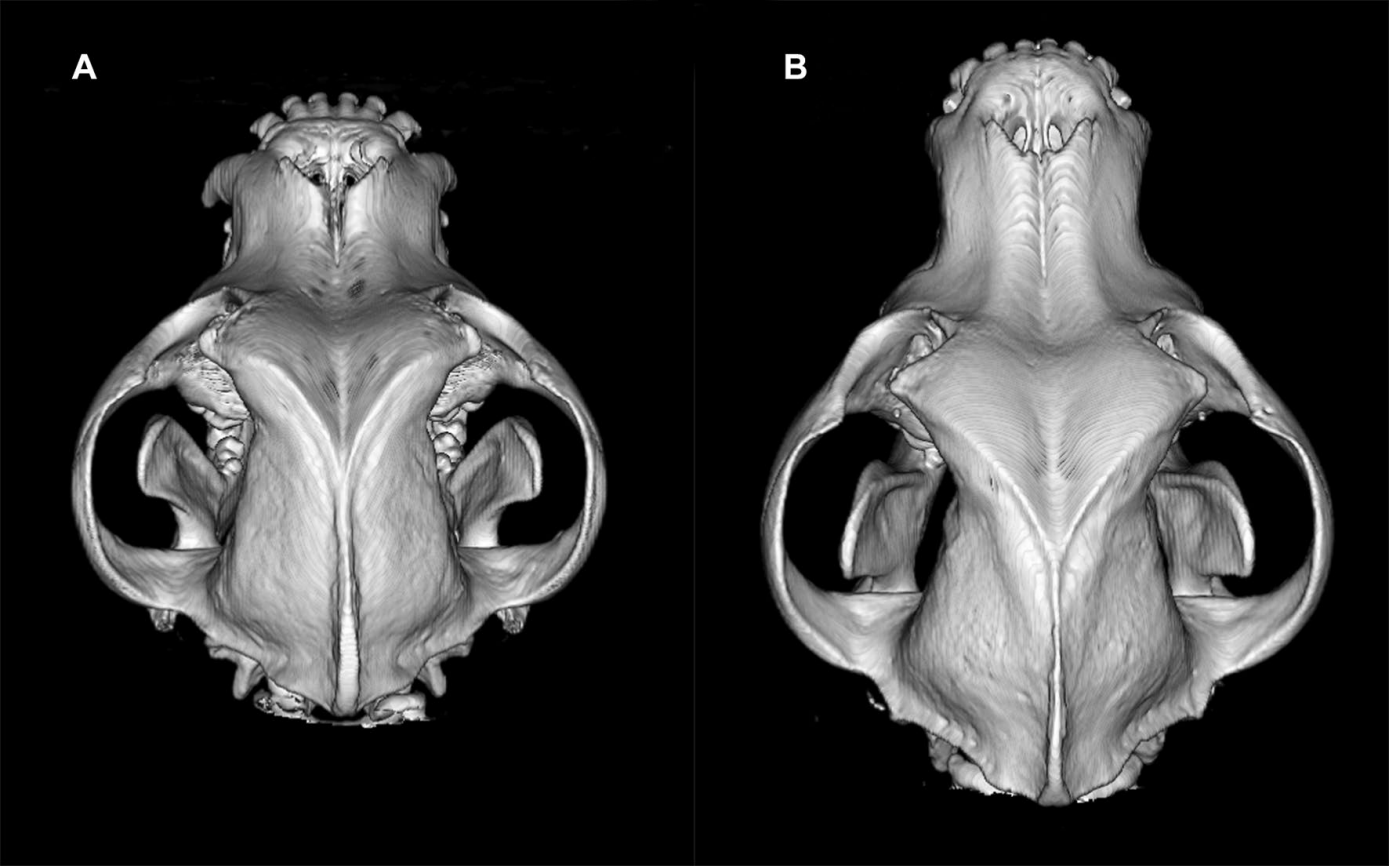
Volume rendering technique (3D) computed tomography images of skulls of two American Staffordshire Terriers. **a** *DVL2* homozygote. **b** *DVL2* wild type. Differences in the skull and facial lengths between genotypes are evident

Of the 11 body measurements, five were associated with *DVL2* genotype: hard palate length, skull base length, the ratio of hard palate length to skull base length, facial index (Fig. 3) and soft palate height. Additionally, the sex of the dog was associated with body weight, height at withers, radius length and hard palate length. Finally, soft palate height was associated with the bodyweight of the dog, with a thicker soft palate correlating with higher body weight.

**Fig. 4:**
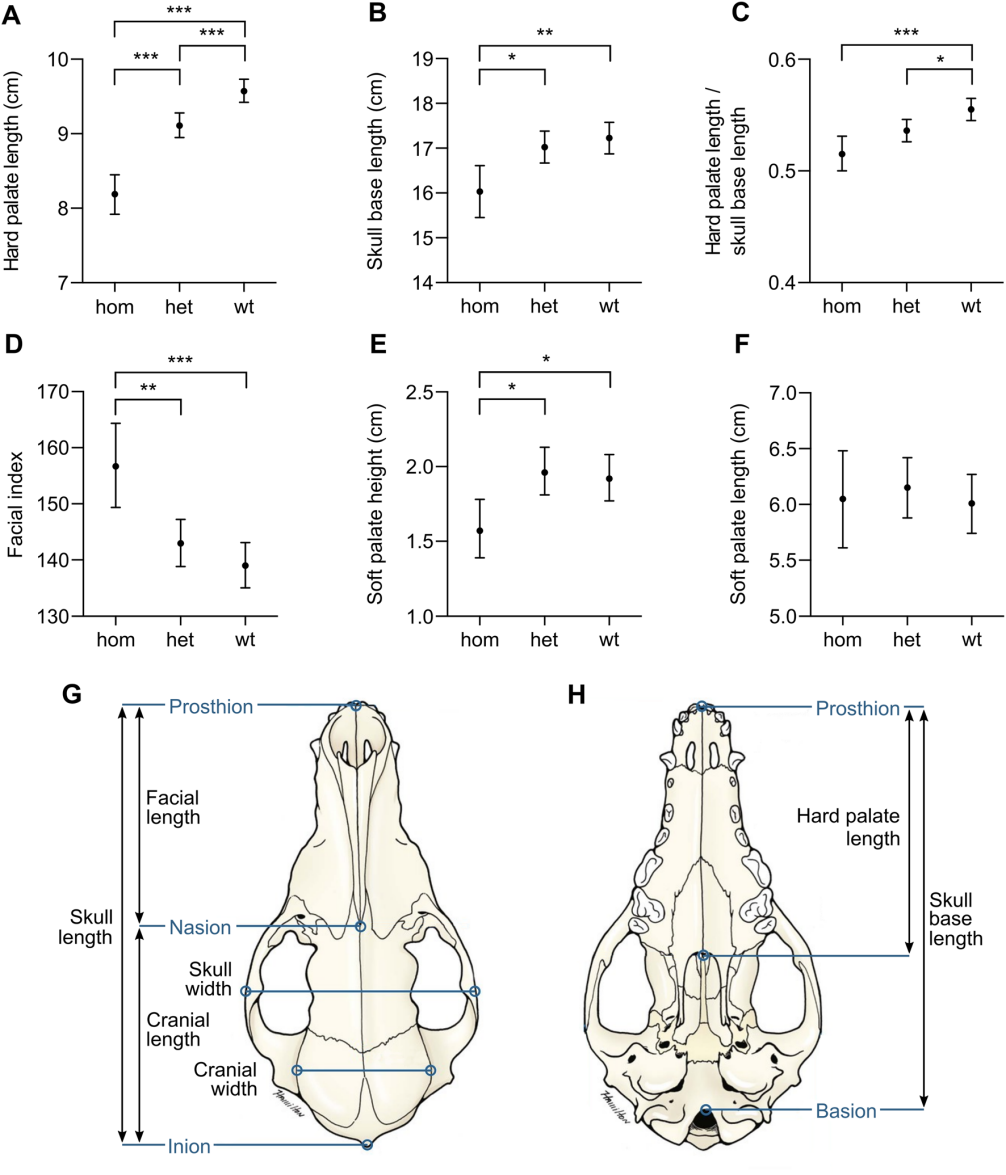
Association of *DVL2* genotypes with body measurements traits, and schematic representation of a dog skull. Asterisks *, ** and *** indicate the significance of *p* < 0.05, *p* < 0.01 and *p* < 0.001, respectively. **a** The length of the hard palate differed between all genotypes. **b** The length of the skull base differed between homozygotes and other genotypes. **c** The ratio of hard palate length to skull base length was smaller in non-wild-type than wild-type dogs. **d** Facial index was higher in homozygotes than other genotypes. **e** The height of the soft palate differed between homozygotes and other genotypes. **f** Genotype was not associated with the length of the soft palate. **g** Dorsal view of a dog skull with craniometrics points. **h** Ventral view of a dog skull with craniometric points Images **g** and **h** are adapted from Miller’s Anatomy of the Dog (4th edition) by Evans H and de Lahunta A, p. 86–87, 2012, Elsevier Health Sciences. Copyright Elsevier (2013). Reprinted with permission

The *DVL2* genotype was significantly associated with hard palate length: homozygotes had a shorter hard palate than heterozygotes (*Z* = − 5.9, df = 1, *P* < 0.0001) and wild-type dogs (*Z* = − 8.8, df = 1, *P* < 0.0001) and heterozygotes had a shorter hard palate than wild types (*Z* = − 4.0, df = 1, *P* = 0.007; Fig. 4a). However, the length of the soft palate did not differ between genotypes (*F* = 0.28, df = 2, *P* = 0.80; Fig. 4f; see pairwise comparisons in Suppl. Table 17). Skull base was shorter in homozygotes than in heterozygotes (*Z* = − 2.9, df = 1, *P* = 0.014) and wild-types (*Z* = − 3.4, df = 1, *P* = 0.003; Fig. 4b). The ratio of hard palate length to skull base length was also lower in homozygotes (*Z* = − 4.2, df = 1, *P* = 0.0003) and heterozygotes (*Z* = − 2.6, df = 1, *P* = 0.028) than in wild types (Fig. 4c). Furthermore, the facial index was higher in homozygotes than in heterozygotes (*Z* = 3.2, df = 1, *P* = 0.006) and wild types (*Z* = 4.2, df = 1, *P* = 0.0003; Fig. 4d). Finally, soft palate height was smaller in homozygotes than in heterozygotes (*Z* = − 3.0, df = 1, *P* = 0.010) and wild types (*Z* = − 2.6, df = 1, *P* = 0.028; Fig. 4e). ANOVA tables are found in Online material 3, Supplementary tables 9–11 and all pairwise contrasts between genotypes in Online material 3, Supplementary tables 12–22.

## Discussion

We screened the Robinow-like syndrome associated *DVL2* variant in 15 breeds, consisting of mainly Bulldog and Pit Bull type dogs as well as Tibetan dogs, to better understand the distribution of the deletion allele in canine populations. Like Mansour et al. (2018), we found that the variant is fixed in French Bulldogs and English Bulldogs. Likewise, all Boston Terriers in our study were homozygous, although a 94% homozygote frequency was observed by Mansour et al. (2018). Additionally, we found the allele in American Staffordshire Terriers, Staffordshire Bull Terriers, Dogues de Bordeaux, American Bulldogs, and Olde English Bulldogges. In ASTs and SBTs, some homozygous individuals were found, which enabled the investigation of the phenotype-genotype correlation to dissect the specific effects of the *DVL2* variant.

To elucidate the quantitative and qualitative effects of the *DVL2* deletion allele on canine morphology, we recruited 19 ASTs to a full-body CT examination and retrospectively assessed radiographic images of 23 dogs. We found that all examined homozygotes had variable caudal vertebral malformations, which is consistent with previous results; however, the total number of caudal vertebrae was not reduced and the tail was not as completely malformed and fused as in English Bulldogs, French Bulldogs and Boston Terriers (Mansour et al. 2018). Additionally, one homozygous dog had sacral vertebral malformations, but lumbar or thoracic abnormalities were not detected in any dogs, including the German Shepherd Dog × French Bulldog crosses, and the shape of the vertebrae were similar in all dogs. This is in sharp contrast to English Bulldogs, French Bulldogs and Boston Terriers, in which previous reports have found a high prevalence of vertebral malformations (Moissonnier et al. 2011; Guevar et al. 2014; Gutierrez-Quintana et al. 2014; Ryan et al. 2017). Our results indicate that either the deletion does not result in thoracic malformations or their penetrance is more variable than the previously estimated 45–100% (Mansour et al. 2018). The incomplete penetrance might also reflect the complex role of *DVL2* in skeletal development, as vertebral malformations were similarly not fully penetrant in Dvl2^−/−^ mice, where 90% of embryos exhibited abnormal vertebral bodies and 25% of surviving offspring had kinked tails (Hamblet et al. 2002). Conversely, caudal vertebral malformations were also present in heterozygous and wild-type dogs, and the allele was not found in King Charles Spaniels, which together indicate that besides the *DVL2* allele, there are additional, still unknown variants that cause tail kinks in dogs. This is further supported by studies in mice, where kinked tail (MP:0000585) is associated with more than 370 genotypes in different strains (Smith and Eppig 2009; Bult et al. 2019).

In addition to the caudal morphological anomalies, we found that the *DVL2* deletion significantly affects several cephalometric measurements. Specifically, a shorter hard palate and skull base length, a smaller ratio of hard palate length to skull base length, and a higher facial index were associated with one or two *DVL2* deletion alleles, which together confirm that the variant results in a more brachycephalic phenotype (Fig. 1). In contrast, the length of the soft palate was not associated with genotype, indicating that it is of similar length in all genotypes despite the reduced facial skeleton length, which results in a mismatch in proportions of the facial skeleton and soft tissues of the oral cavity, i.e., an elongated soft palate. Importantly, elongated soft palate, together with stenotic nares, is a primary abnormality in brachycephalic obstructive airway syndrome (BOAS) (Stockard 1941; Harvey 1989). Shortening of the skeletal muzzle results in a cramming effect, with the “excess” soft tissues interfering with airflow and partially blocking the nasopharynx and the larynx during respiration (Harvey 1989). Stenotic nares further increase airflow resistance and impair thermoregulation (Knecht 1979; Oechtering et al. 2010). As a result, affected individuals have clinical signs such as respiratory distress, dyspnea, and heat and exercise intolerance, and surgery of the soft palate and nares as well as secondary changes, such as laryngeal collapse, is often needed to reduce airway obstruction and improve quality of life (Packer and Tivers 2015).

Our results strongly indicate brachycephaly and elongated soft palate in dogs with the *DVL2* deletion allele and suggest that additional risk for BOAS may be conferred by the variant. However, our cohort size was very small, with only three homozygotes, and the allele acted with variable mechanisms, including incompletely dominant (hard palate length), fully dominant (ratio of hard palate length to skull base length) or recessive (skull base length, facial index and soft palate height), depending on the measurement. Thus, our results need to be confirmed in a larger cohort before a definitive mode of inheritance can be determined for each trait.

In addition to altered facial proportions, mesomelic limb shortening is a typical feature in human Robinow patients (White et al. 2018). In contrast, while our data did show a slight trend between *DVL2* genotype and a shorter radius as well as the lower height at withers, the results were not statistically significant (Online Material 3, Suppl. tables 13–14). These body proportions will also need to be studied in a larger cohort to confirm whether mesomelic dwarfism is a part of the phenotype in *DVL2* homozygous dogs.

As an additional discovery, we found that two out of three homozygous dogs in the CT study had a mild heart murmur, and one of them had a previously confirmed diagnosis of aortic stenosis. During organogenesis, aortic and pulmonary valves arise from endocardial cushions with the involvement of cardiac neural crest cells, and these cell populations are also involved in the septation of the common outflow tract into the aorta and pulmonary trunk (Waldo et al. 1998; Nakajima et al. 2000; Jiang et al. 2000; Délot 2003; Brown and Baldwin 2006; Hutson and Kirby 2007). Notably, Dvl2^−/−^ mice exhibit various outflow tract malformations, including double outlet right ventricle, transposition of the great arteries, and persistent truncus arteriosus, due to a signaling defect in the cardiac neural crest (Hamblet et al. 2002). Based on this common developmental origin of the outflow tract and semilunar valves, it can be hypothesized that the *DVL2* deletion may be involved in cardiovascular anomalies in homozygous dogs. However, this suggestion is speculative and warrants further research, especially since pulmonary stenosis and aortic stenosis are among the most common congenital heart defects in English Bulldogs, French Bulldogs and ASTs (Brambilla et al. 2020).

Screw tail is a breed characteristic in Boston Terriers, French Bulldogs and English Bulldogs, and they are fixed for the *DVL2* deletion. In contrast, caudal vertebral anomalies are undesired and selected against in ASTs, SBTs and Dogues de Bordeaux, which is paralleled by the lower allele frequency (18.9–37.0%) in these breeds. Notably, the genotypes observed are in Hardy–Weinberg equilibrium in ASTs but not in SBTs despite a large number of dogs. Based on the frequency of the *DVL2* allele in SBTs, we would have expected to find more deletion homozygotes (observed = 1, expected = 6.6, *P* < 0.015). Whether this reflects a sampling bias or some other phenomenon, e.g. removal of homozygous dogs from the population, is currently unknown to us.

Altogether, the quantitative and qualitative morphological changes confirm that the *DVL2* deletion results in a syndromic phenotype resembling Robinow syndrome in humans, supporting the previous hypothesis of the canine Robinow-like syndrome. Still, the features on the spectrum of human Robinow syndrome are not influenced solely by *DVL2* in dogs: as an example, the brachycephalic, short-limbed and vertebral phenotypes are more extreme in English Bulldogs, French Bulldogs and Boston Terriers due to selective breeding. In general, many morphological traits in dogs are genetically complex and affected by multiple variants, with their frequencies influenced by differential selective pressures in different breeds. Examples of such variants include the brachycephaly associated *SMOC2* and *BMP3* variants and chondrodysplasia-associated *FGF4* retrogenes on chromosomes 12 and 18 (Parker et al. 2009; Schoenebeck et al. 2012; Marchant et al. 2017; Brown et al. 2017), and there are likely more, yet undiscovered variants. Additionally, it is notable that in zebrafish, zygotic dvl2 mutants are relatively normal compared to wild-type individuals, whereas maternal-zygotic mutants exhibit severe craniofacial defects resembling a “bulldog facial phenotype” (Xing et al. 2018). To our knowledge, the possible maternal effect of *DVL2* in mammals has not been studied. Thus, it can be hypothesized that Robinow-like syndrome could also be more extensive in dogs with deletion homozygous dams, which is always the case in breeds where the allele is fixed. However, further research is needed to test whether our hypothesis is correct.

In summary, our study strengthens the role of the *DVL2* variant in the brachycephalic phenotype and caudal vertebral anomalies, and suggests that other conditions may be linked to canine Robinow-like syndrome, including BOAS and congenital heart defects. Kinked tail is an undesired trait in non-screw tail breeds and our study shows that screening of the *DVL2* variant could help to redesign breeding plans to reduce unwanted *DVL2*-related anomalies and improve canine welfare.

## Supplementary Information

Below is the link to the electronic supplementary material.

Online material 1. Findings and measurements in 19 CT examined American Staffordshire Terriers (XLSX 30 KB)

Online material 2. Findings and measurements in 23 radiographed American Staffordshire Terriers (XLSX 281 KB)

Online material 3. Results of statistical analyses of 11 body measurements, ratios and indices (XLSX 13 KB)

## Data Availability

All data generated or analysed during this study are included in this published article and its supplementary information files.
